# FGF21 Attenuated LPS-Induced Depressive-Like Behavior *via* Inhibiting the Inflammatory Pathway

**DOI:** 10.3389/fphar.2020.00154

**Published:** 2020-02-28

**Authors:** Xue Wang, Liyun Zhu, Jian Hu, Ruili Guo, Shasha Ye, Fei Liu, Dongxue Wang, Yeli Zhao, Aiping Hu, Xiaojie Wang, Kaiming Guo, Li Lin

**Affiliations:** ^1^ School of Pharmaceutical Sciences, Wenzhou Medical University, Wenzhou, China; ^2^ Engineering Laboratory of Zhejiang Province for Pharmaceutical Development of Growth Factors, Biomedical Collaborative Innovation Center of Wenzhou, Wenzhou, China

**Keywords:** major depressive disorder, depressive-like behavior, FGF21, inflammation, NF-κB signaling pathway

## Abstract

Major depressive disorder is a serious neuropsychiatric disorder with high rates of recurrence and mortality. Many studies have supported that inflammatory processes play a central role in the etiology of depression. Fibroblast growth factor 21 (FGF21), a member of the fibroblast growth factors (FGFs) family, regulates a variety of pharmacological activities, including energy metabolism, glucose and lipid metabolism, and insulin sensitivity. In addition, recent studies showed that the administration of FGF21, a regulator of metabolic function, had therapeutic effects on mood stabilizers, indicating that FGF21 could be a common regulator of the mood response. However, few studies have highlighted the antidepressant effects of FGF21 on lipopolysaccharide (LPS)-induced mice, and the anti-inflammatory mechanism of FGF21 in depression has not yet been elucidated. The purpose of the current study was to determine the antidepressant effects of recombinant human FGF21 (rhFGF21). The effects of rhFGF21 on depression-like behaviors and the inflammatory signaling pathway were investigated in both an LPS-induced mouse model and primary microglia *in vitro*. The current study demonstrated that LPS induced depressive-like behaviors, upregulated proinflammatory cytokines, and activated microglia in the mouse hippocampus and activated the inflammatory response in primary microglia, while pretreatment with rhFGF21 markedly improved depression-like behavior deficits, as shown by an increase in the total distance traveled and number of standing numbers in the open field test (OFT) and a decrease in the duration of immobility in the tail suspension test (TST) and forced swimming test (FST). Furthermore, rhFGF21 obviously suppressed expression levels of the proinflammatory cytokines interleukin-1β (IL-1β), tumor necrosis factor-α (TNF-α), and interleukin-6 (IL-6) and inhibited microglial activation and the nuclear factor-κB (NF-κB) signing pathway. Moreover, coadministration of rhFGF21 with the fibroblast growth factor receptor 1 (FGFR1) inhibitor PD173074 significantly reversed these protective effects, indicating that the antidepressant effects of rhFGF21 occur through FGFR1 activation. Taken together, the results of the current study demonstrated for the first time that exogenous rhFGF21 ameliorated LPS-induced depressive-like behavior by inhibiting microglial expression of proinflammatory cytokines through NF-κB suppression. This new discovery suggests rhFGF21 as a new therapeutic candidate for depression treatment.

## Introduction

Major depression disorder (MDD), a mood disorder characterized by the symptoms of a persistent feeling of sadness, loss of interest, and worthlessness, affects approximately 300 million people worldwide (Zhang et al., 2019). A total of 0.8 million patients with depression commit suicide annually (Lee et al., 2019). There are some clinical antidepressants on the market, such as norepinephrine reuptake inhibitors, selective serotonin reuptake inhibitors, and monoamine oxidase inhibitors; however, only one-third of patients respond to these therapeutics (Li, 2019), which are often associated with numerous side effects and a high risk of relapse after drug withdrawal, such as the 61.8% relapse rate in the case of fluoxetine (Andrews et al., 2012; Lee et al., 2019). Therefore, further study to develop novel effective antidepressants is urgently needed.

Several hypotheses to explain the pathology of depression, including glutamatergic excitotoxicity, monoamine system impairment, hypothalamic-pituitary-adrenal axis dysfunction, neuroinflammation, and neural plasticity and neurogenesis disruption, have emerged (Taniguti et al., 2019). Among these hypotheses, inflammatory processes have been suggested to be involved in the etiology of depression in many studies (Domingues et al., 2018). Increasing amounts of preclinical and clinical research have shown that proinflammatory cytokines might contribute to depression. The proinflammatory cytokines tumor necrosis factor-α (TNF-α) and interleukin-6 (IL-6) play critical roles in the process of inflammation and can induce depressive disorders (Wright et al., 2005; Guo et al., 2019). The levels of IL-6 and TNF-α were reported to be increased in the blood and cerebrospinal fluid of MDD patients (Syed et al., 2018). Escherichia coli lipopolysaccharide (LPS), a commonly used proinflammatory endotoxin, can trigger microglial activation and induce immune activation and behavioral changes that are similar to the clinical symptoms of human depression (Adzic et al., 2015; Taniguti et al., 2019). Therefore, mice were administered LPS to serve as a model of central nervous system (CNS) inflammation and induce depression-like behaviors. Nuclear factor-κB (NF-κB), a major transcription factor, is involved in the activation of an exceptionally large number of genes in response to inflammation. Once stimulated by LPS, NF-κB translocates to the nucleus and regulates the expression of inflammatory cytokines such as TNF-α, interleukin-1β (IL-1β), and IL-6 (Lin et al., 2007; Domingues et al., 2018; Muhammad et al., 2019).

Fibroblast growth factor 21 (FGF21), a member of the fibroblast growth factor (FGF) family, is mainly expressed in the liver and functions as a hormone. After its secretion, FGF21 regulates a variety of pharmacological activities; whole-body energy metabolism, especially glucose and lipid metabolism; and insulin sensitivity (Xu et al., 2009; Gaich et al., 2013; Pan et al., 2018; Li, 2019). In addition, recent studies showed that the administration of FGF21, a regulator of metabolic function, had a therapeutic effect on mood stabilizers (Chang et al., 2018). In bipolar disorder (BD) patients, the FGF21 level was significantly enhanced after valproate treatment; however, there was no significant difference between the FGF21 levels of healthy control and BD patients, indicating that FGF21 may be a common regulator of the mood response (Chang et al., 2018). Moreover, a previous study reported a significant negative association between cerebrospinal fluid FGF21 levels and Beck Depression Inventory (BDI) scores in male Chinese subjects, but not in female (Liu et al., 2017). These results further demonstrate that FGF21 plays a role in mood regulation; however, the mechanism by which FGF21 mediates mood disorders is not clear.

In the current study, the effects of recombinant human FGF21 (rhFGF21) on the depression-like behaviors of LPS-induced mice models were evaluated. The NF-κB signaling pathway is involved in inflammatory events. Once stimulated by LPS, NF-κB is phosphorylated and activated, and phosphorylated NF-κB translocates into the nucleus and binds a consensus sequence in targeted genes to regulate the expression of inflammatory cytokines such as TNF-α, IL-6, and IL-1β (Yang M. et al., 2018). Therefore, we speculated that the anti-depressive effect of rhFGF21 is mediated by NF-κB signaling pathway regulation. To confirm this hypothesis, we evaluated the expression levels of inflammatory cytokines in the hippocampus of an LPS-induced depression-like model and in primary microglia. Additionally, the NF-κB signaling pathway was analyzed to further explore the possible underlying mechanism of the antidepressant effects of rhFGF21.

## Materials and Methods

### Reagents and Antibodies

rhFGF21 was supported from Key Laboratory of Biopharmaceutical, School of Pharmaceuticals Sciences, Wenzhou Medical University, that is produced and purified from Escherichia according to the reference (Wang et al., 2010). FGFR1 inhibitor PD173074 was purchased from Selleckchem (Houston, TX, USA). LPS was obtained from Sigma (Sigma-Aldrich, St Louis, MO). The following primary antibodies applied were purchased from Abcam (Cambridge, MA, USA): anti-FGFR1 (No. ab824), anti-p-FGFR1 (No. ab59194), and anti-β-actin (No. ab8227); anti-NF-κB (No. 8242) and anti-p-NF-κB (No. 3033s) were purchased from Cell Signalling Technology (Danvers, MA, USA); anti-BDNF (No. BS9896M) and anti-Iba1 (No. 019-19741) were purchased from Bioworld technology (Louis Park, MN, USA) and FUJIFILM Wako Pure Chemical Corporation (Osaka, Japan), respectively. The secondary antibodies used in this study were goat anti-rabbit IgG H&L (HRP) (No. ab6721) and Alexa Fluor ^®^488-conjugated donkey anti-rabbit (No. ab150073) purchased from Abcam (Cambridge, MA, USA).

### Animals

The experiments were conducted in male C57BL/6N mice (20–25 g), which were purchased from the Animal Center of the Chinese Academy of Sciences (Beijing, China). The animal use and care protocol conformed to the Guide for the Care and Use of Laboratory Animals from the National Institutes of Health and was approved by the Animal Care and Use Committee of Wenzhou Medical University.

### Drug Administration and Animal Experimental Procedures

**Figure 1:**
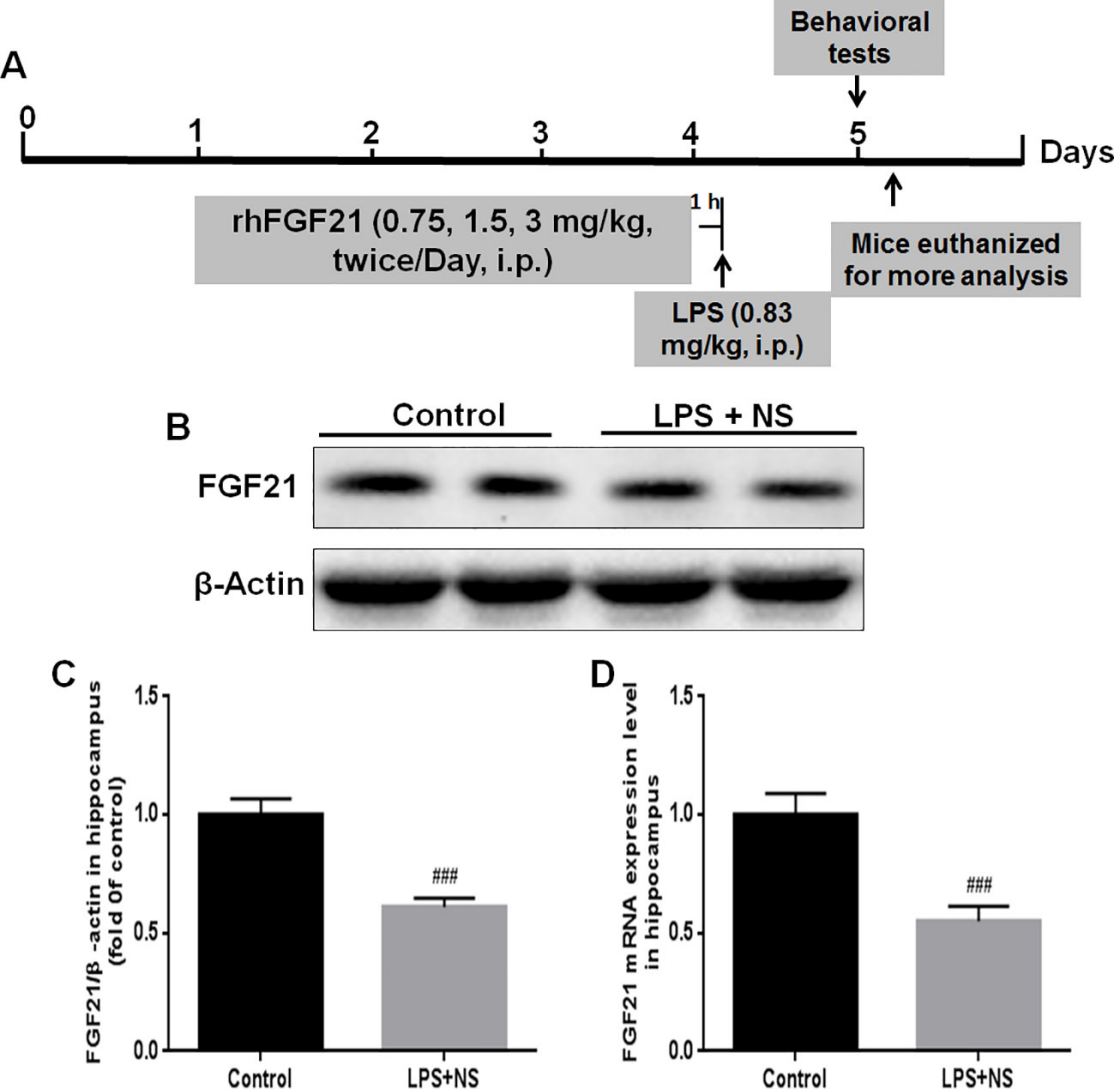
Endogenous FGF21 expression level in the hippocampus of LPS-induced mice was reduced. **(A)** Schematic illustration of animal experimental procedures showing the duration of the lipopolysaccharide (LPS) and/or exogenous rhFGF21 administration in adult mice and analysis. **(B)** Representative bands of endogenous FGF21 expression detected by western blot. **(C)** Densitometric analysis for the protein expression of FGF21. **(D)** Endogenous FGF21 mRNA expression level in the hippocampus of LPS-induced mice. Data are means ± SEM (n = 5). ^###^p < 0.001 compared to control group.

The experimental design and protocol used for the animal experiment in this study are illustrated in **Figure 1A**. The mice were divided into three groups randomly; (1) the control vehicle (normal saline, NP) administered group; (2) the LPS + NP treated group; (3) the LPS + rhFGF21 treated group. Mice were pretreated intraperitoneally (i.p.) with vehicle or rhFGF21 (0.75, 1.5, and 3 mg/kg) twice daily for three consecutive days. These doses were chosen because it has previously been shown that rhFGF21 with the dose of 1.5 mg/kg dramatically attenuated locomotor function deficits in mice (Jiang et al., 2018). One hour after the last rhFGF21 administration on day 3, the mice were treated i.p. with LPS (0.83 mg/kg) dissolved in sterile saline. The concentration of LPS was based on the results of previous studies (Tomaz et al., 2014; Taniguti et al., 2019; Wang et al., 2019a; Wang et al., 2019b). The animals were subjected to following behavioral tests 24 h after LPS. Then, the animals were deeply anesthetized with isoflurane and euthanized by decapitation. The hippocampal tissue was rapidly removed and stored at -80°C until assays. NF-κB, iNOS, and brain-derived neurotrophic factor (BDNF) protein levels were assessed by western blot analysis (n = 5). Levels of the proinflammation factors IL1-β, TNF-α, and IL-6 were determined by RT-PCR (n = 5). To detect Iba1, NF-κB, and FGFR1 by immunofluorescence localization (n = 4), mice were deeply anesthetized and subjected to cardiac perfusion with saline followed by perfusion with 4% paraformaldehyde (PFA). Their brains were rapidly removed and post-fixed in 4% PFA overnight for further immunofluorescence analysis (**Figure 1A**).

### Behavioral Assay

Depression behavior was monitored by increased duration of immobility in the forced swim test (FST) and decreased sucrose preference; meanwhile, sickness behavior was measured by body weight loss, reduced food intake, and locomotor activity. It has been reported that LPS-induced depression-like behaviors can be dissociated from sickness from 24 h after LPS administration (Walker et al., 2013; Walker et al., 2019). The sickness responses and behavior (piloerection, ptosis and lethargy) was usually measured during peak period of sickness at 6 h after LPS administration (Silverman et al., 2013; Walker et al., 2019). In our behavioral experiment, we forced on examining the effects of rhFGF21 on depression-like behavior measured at 24 h after LPS, which include FST and locomotor activity monitored by tail suspension test (TST) and open field test (OFT) (n = 9) (Yu et al., 2019).

The TST, a behavioral test used to evaluate depressive-like behavior in animals, was conducted as previously described (Steru et al., 1985; Lee et al., 2019) with slight modifications. The TST was carried out in a soundproof box (40 × 20 × 60 cm) with cameras on the side and bottom of the box. Briefly, a string was fixed to each mouse with tape 1 cm from the tip of its tail. The mice were 20 cm above the ground, and the test was carried out for 6 min. The immobility time was recorded during 5 min of a 6-min observation period.

The FST is a common behavior test used to evaluate depression-like behavior. The immobility time after each mice ceased struggling and remained floating motionless in the water was recorded. A decrease in the duration of immobility is indicative of an antidepressant-like effect (Porsolt et al., 1977). In this test, mice were individually placed into a cylinder (25 cm in height, 10 cm in diameter) containing water at a depth of 19 cm of water at 25 ± 1°C. After a habituation period (2 min), the total amount of time each animal remained immobile during a 6-min session was scored as the immobility time, as described previously (Taniguti et al., 2019).

The OFT, a method used to evaluate the autonomous behavior, inquiry behavior, and tension of experimental animals in new environments, was carried out according to a previous study with few modifications (Ieraci and Herrera, 2006). In brief, mice were individually placed in the center of a black wooden box (50 × 50 × 50 cm) that served as the open field. Locomotion behavior indicated by the number of standing number was recorded for 5 min using a camera and analyzed using an open field experimental video analysis system (Smart 3.0, Panlab SMART video tracking system, Barcelona, Spain). The arena floor was cleaned with a 10% ethanol solution between trials.

### Primary Rat Microglia Culture

Primary rat microglia cultures were prepared from the cerebral cortices of 1–2-day-old neonatal Sprague-Dawley rat pups with mild trypsinization as previously described with minor modifications (Lin et al., 2017). Briefly, after removing the meninges of the brain, the cortical cortices were dissected and cut into small, 2-mm pieces, and tissues were digested with 0.25% trypsin for 30 min at 37°C. The tissues were suspended in DMEM/F12 containing 10% FBS and 1% penicillin-streptomycin and mechanically triturated with a plastic P1000 pipette tip. Then, the mixed cells were passed through a 70-μm nylon mesh cell strainer and plated on 6-well plates or cell culture dishes. After three days, the medium was completely replaced and changed every three days with fresh medium. After approximately 14 days, the mixed cells achieved 90% confluency, and microglia were isolated from mixed glial cultures *via* mild trypsinization according to our previous study (Lin et al., 2017). Mixed glial cultures were incubated with a trypsin solution containing 0.25% trypsin-EDTA for 30 min to detach a layer of cells. Primary microglial cells remained attached to the bottom of the plate and were used for further study.

### Western Blot Analysis

Total protein from brain hippocampal tissue and primary microglia was extracted using protein extraction reagents containing 1% protease and phosphatase inhibitors. Nuclear and cytoplasmic protein was purified using a Nuclear and Cytoplasmic Protein Extraction Kit (Beyotime Biotechnology, Shanghai, China). The protein content of the samples was measured by a BCA Protein Assay Kit (Beyotime Biotechnology, Shanghai, China). An equivalent amount of protein (30 μg) was separated on an SDS-PAGE gel and then transferred onto a Polyvinylidene Fluoride (PVDF) membrane. After being blocked with 5% non-fat milk in Tris-buffered saline (TBS) containing 1% Tween for 2 h at room temperature, the membranes were further incubated with primary antibodies overnight at 4°C (a 1:400 dilution of anti-FGFR1, and 1:1,000 dilution of anti-p-FGFR1, anti NF-κB, anti-p-NF-κB, anti-BDNF, and β-Actin). After three washes with TBST, the membranes were incubated with a 1:10,000 dilution of goat anti-rabbit IgG secondary antibody for 1 h at room temperature. Finally, the immunoreactive protein bands were developed and visualized with an enhanced chemiluminescence (ECL) kit (Biological Industries, Kibbutz Beit-Haemek, Israel), and the band densities were quantified using Image Lab 5.0 software (Bio-Rad, CA, USA).

### Immunofluorescence Analysis

Immunofluorescence analysis was performed to determine the localization of Iba1 in the mice brain hippocampal tissue. In brief, the whole brain was post-fixed by 4% PFA for 12 h, embedded in paraffin, and cut into section (5 µm thick), followed by mounted on slides. Sections were deparaffinized and rehydrated. Then tissue was incubated with 3% H_2_O_2_ for 15 min, followed blocked nonspecific binding in 5% bovine serum albumin (BSA) for 30 min at 37°C. Then sections were treated with primary antibodies anti-Iba1 (1:1,000), NF-κB (1:1,000), FGFR1 (1:1,000) overnight at 4°C, followed by incubation with AlexaFluor 488 donkey anti-rabbit secondary antibody (1:1,000) at 37°C for 1 h. Then, the nuclei were stained with DAPI for 7 min. The immunostained sections were observed and imaged using a Nikon ECLPSE 80i fluorescence microscope (Nikon, Tokyo, Japan). Three indexes of microglia activation (number, soma size, and process length) were measured according to the previous study (Tang et al., 2018). The density of Iba1 positive cells were automatically analyzed by Image-Pro plus 6.0 software (Bethesda MD, USA) at ×10 magnification in a defined area. The area of microglia soma and the microglia process length were measured at ×20 magnification by Image-Pro plus 6.0 software. For each group, at least six representstive images were taken from four mice.

### RNA Extraction and RT-PCR

Quantitative real-time PCR with SYBR Green dye was applied to measure whether rhFGF21 affected the mRNA expression level of pro-inflammation cytokines including IL-1β, TNF-α, and IL-6. Total RNA was extracted from mice brain hippocampal tissue and primary microglia by the RNeasy Mini Kit (Qiagen, Hilden, Germany) according to the manufacturer's instructions. The residual genomic DNA was removed by RQ1 RNase-Free DNase (Promega, Fitchburg, WI, USA), and 1 μg of the total RNA from each sample was applied for complementary DNA (cDNA) synthesis using the PrimeScriptTM RT Reagent Kit (TaKaRa, Kusatsu, Japan). Real-time qRT-PCR was performed in 96-well plates using a quantitative PCR system (CFX ConnectTM Real-Time System, Bio-Rad, CA, USA). Each reaction mixture consisted of 10 μl of the SYBR Green PCR Master Mix Kit (Applied Biosystems, Carlsbad, CA, USA), 2 μl of the forward and reverse primers (5 pmol each), 25 ng of the cDNA, and diethylpyrocarbonate water for a final volume of 20 μl. The oligonucleotide PCR primer pairs are listed in **Table 1**, purchased from Sangon Biotech (Shanghai, China). The cycling program was an initial hold at 95°C for 5 min, followed by 40 cycles of denaturation at 95°C for 30 s, annealing at 62°C for 30 s, and extension at 72°C for 30 s. The mRNA expression levels of the target genes were normalized to the mRNA expression level of the housekeeping gene β-actin, and analyzed by the 2^–ΔΔCT^ method. The results are expressed as the means ± SEMs of duplicate samples from three independent experiments.

**Table 1:** Primers' sequences used for real-time PCR analysis.

Gene	Sequence 5'-3'	Amplification length (bp)
FGF21	CGACTGCTGCTGGCTGTCTTC	135
	GGCTTCAGTGTCTTGGTCGTCATC	
IL-1β	AAGCCTCGTGCTGTCGGACC	140
	TGAGGCCCAAGGCCACAGGT	
TNF-α	CAAGGGACAAGGCTGCCCCG	109
	GCAGGGGCTCTTGACGGCAG	
IL-6	AGAAGGAGTGGCTAAGGACCAA	101
	AACGCACTAGGTTTGCCGAGTA	
β-actin	CACTGCAAACGGGGAAATGG	198
	TGAGATGGACTGTCGGATGG	

### Statistical Analysis

All data are presented as mean ± SEM from at least three independent experiments. Statistical analyses were performed using GraphPad Prism 7 (GraphPad software Inc., San Diego, CA, USA). Statistical significance between groups was determined by one-way analysis of variance (ANOVA) followed by Turkey's test using. P < 0.05 was considered statistically significant.

## Results

### Endogenous FGF21 Was Reduced in the Hippocampus of the LPS-Induced Mouse Brain

FGF21 plays an important role in mood regulation, and its levels were significantly increased in BD patients after treatment with the antidepressant drug valproate (Chang et al., 2018). Therefore, we detected the endogenous expression levels of both FGF21 protein and FGF21 mRNA. LPS treatment significantly decreased the endogenous FGF21 protein (**Figures 1B, C** **)** and mRNA (**Figure 1D**) expression levels compared to those in saline-treated mice (control group).

### Exogenous rhFGF21 Administration Alleviated Depressive-Like Behavior Induced by LPS

**Figure 2:**
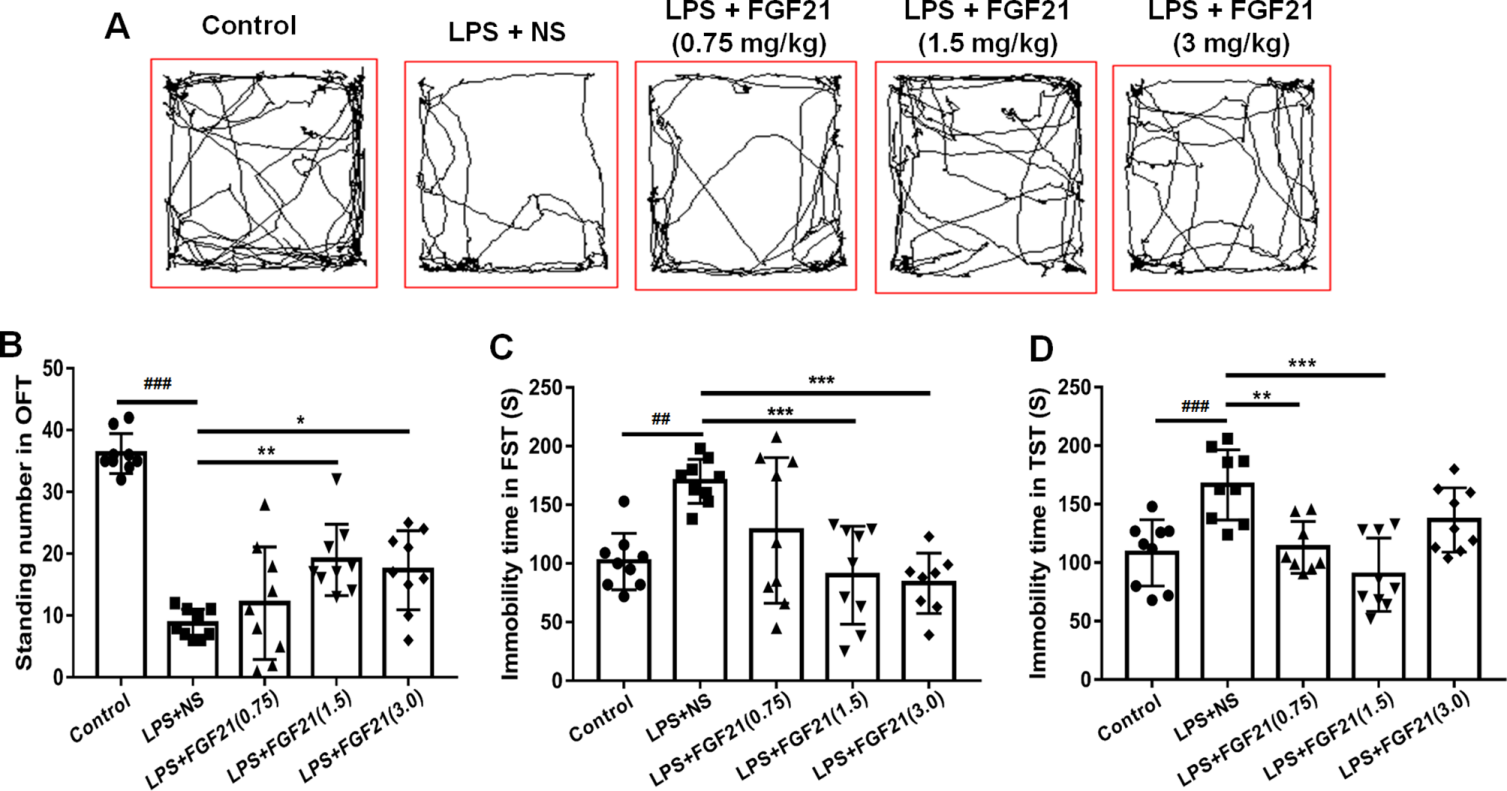
Exogenous rhFGF21 (0.75, 1.5, and 3 mg/kg) administration alleviated depression-like behaviors in LPS-induced mice. **(A)** Total distance traveled and line crossing in the open filed. **(B)** Standing number in the open filed. **(C)** Immobile time during the FST. **(D)** Immobile time during the TST. Data are means ± SEM (n = 9). ^*^p < 0.05, ^**^p < 0.01, ^***^p < 0.001, ^###^p < 0.001, ^##^p < 0.01.

**Figure 3:**
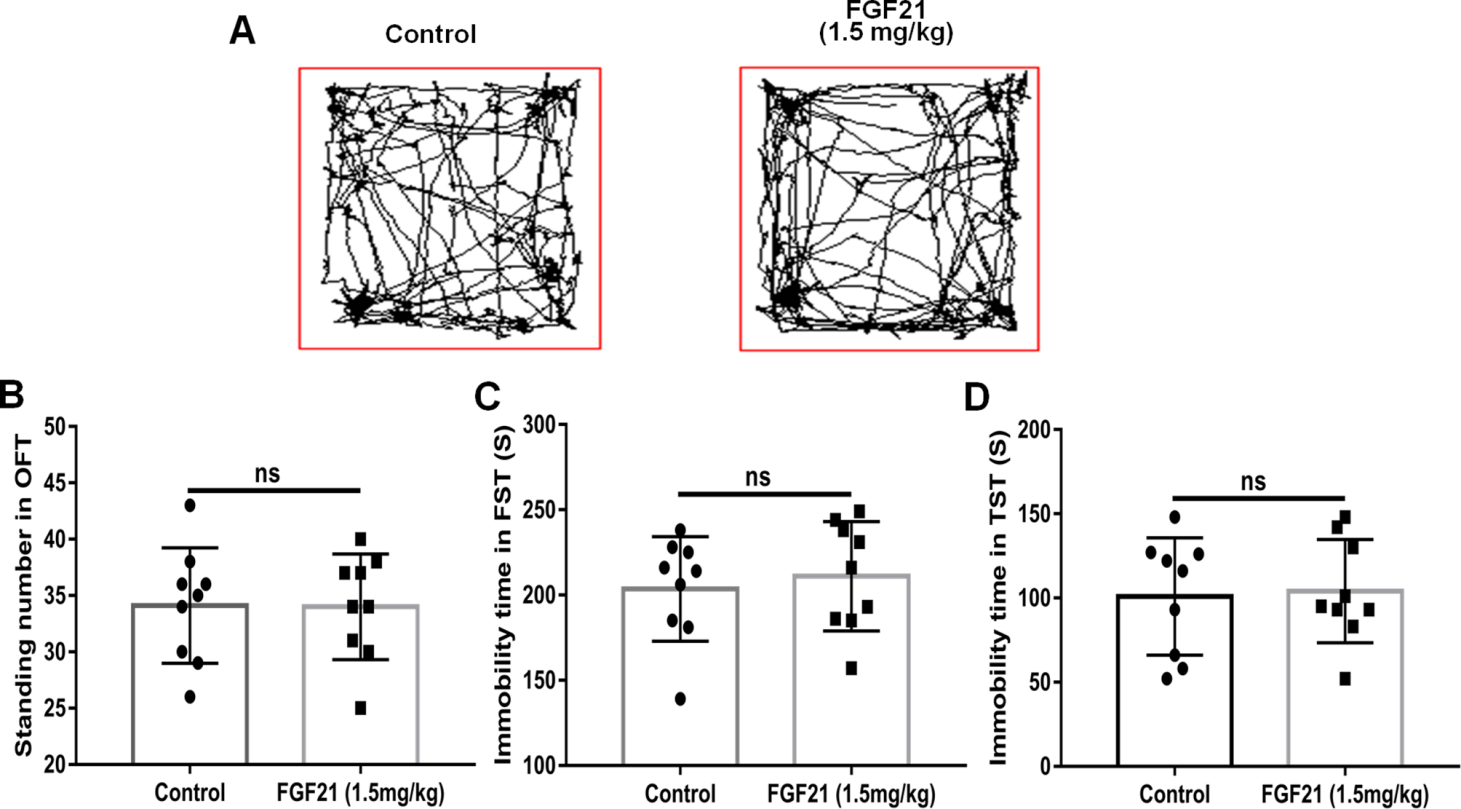
Exogenous rhFGF21 administration (1.5 mg/kg) did not change the behavior of normal mice. **(A)** Total distance traveled and line crossing in the open filed. **(B)** Immobile time during the FST. **(C)** Standing number in the open filed. **(D)** Immobile time during the TST. Data are means ± SEM (n = 9). ns, no significant difference.

To evaluate the effects of rhFGF21 on depressive-like behavior induced by LPS, the OFT, FST, and TST were performed. Mice were pretreated with saline or rhFGF21 (0.75, 1.5, and 3 mg/kg, i.p.) for three days, followed by LPS administration (0.83 mg/kg, i.p.). In the OFT, a general measure of curiosity and detective behavior, the total distance traveled, line crossings, and number of standing events are used to assess locomotor activity. LPS significantly suppressed the total distance traveled and number of standing events compared with those in the control group (**Figures 2A, B** **)**. Pretreatment with different concentrations of rhFGF21 significantly improved the decreased total distance traveled (**Figure 2A**) and number of standing events caused by LPS treatment (**Figure 2B**). In the FST, a putative indicator of behavioral despair, LPS administration markedly increased the mouse immobility time compared with that of the control group, and rhFGF21 treatment significantly decreased the increased immobility time induced by LPS (**Figure 2C**). In the TST, a classic method to assess mood, the time taken until the mouse remained immobile was measured. LPS significantly increased the immobility time during suspension, indicating that LPS induced depression-like behaviors. rhFGF21 administration significantly reduced immobility compared with that in the LPS-treated group (**Figure 2D**). As shown by behavioral analyses, pretreatment with rhFGF21 at doses of 0.75, 1.5, and 3 mg/kg significantly alleviated LPS-induced depression behavioral deficits; however, 1.5 and 3 mg/kg rhFGF21 had a greater effect than 0.75 mg/kg rhFGF21. Therefore, rhFGF21 at a dose of 1.5 mg/kg was applied in further studies. Moreover, rhFGF21 without LPS administration did not change the total distance traveled, or number of standing events in the OFT or the immobility time of the mice in the FST and TST (**Figures 3A–D**). These results demonstrate that rhFGF21 improved depression-like behaviors induced by LPS and was effective only in disease conditions.

### Exogenous rhFGF21 Administration Reduced LPS-Induced Microglial Activation

**Figure 4:**
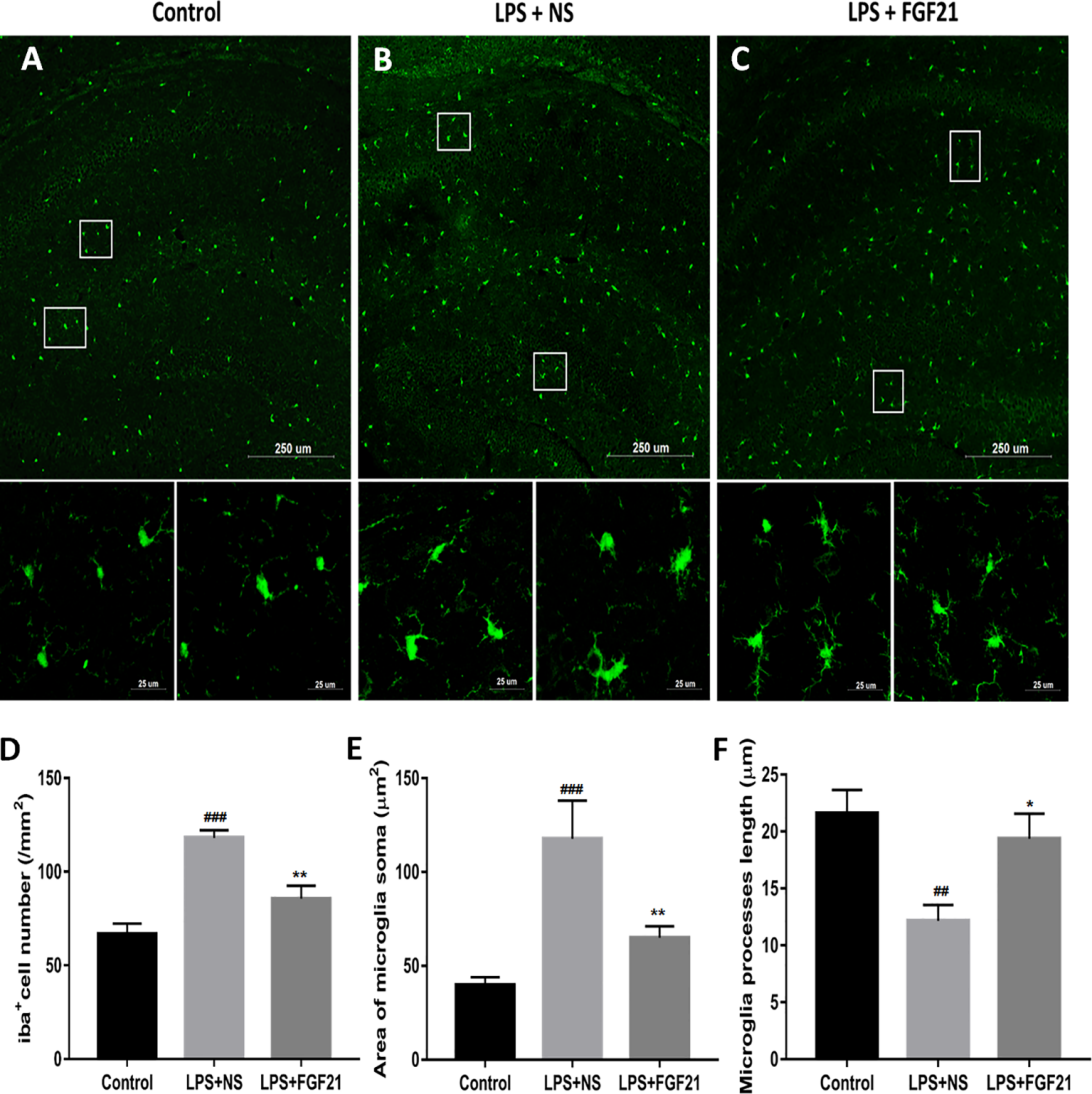
Exogenous rhFGF21 administration reduced microglial activation in LPS-induced mice hippocampus. **(A)** Representative images of microglia in control group. **(B)** Representative images of microglial activation in LPS-induced group. **(C)** Representative images of microglial activation in rhFGF21 pretreated LPS-induced group. **(D)** Quantification of microglial cell number per square millimeter. **(E)** Quantification of microglial area soma. **(F)** Quantification of microglial process length. Data are means ± SEM (n = 4, 3–4 tissue sections per animal). ^##^p < 0.01, ^###^p < 0.001 compared to control group, ^*^p < 0.05, ^**^p < 0.01 compared to LPS-induced group.

Microglia, the innate immune cells in the brain, responds to inflammation (Lenz and Nelson, 2018). In depressed patients who committed suicide, microglial activation was observed, indicating that microglia play an important role in neuroinflammation in the brain during the pathogenesis of depression (Brites and Fernandes, 2015). The transfer of microglia from a ramified status to an activated status is marked by reduced process length and swollen soma (Kettenmann et al., 2011; Tang et al., 2018). To further investigate the antidepressant effect of rhFGF21 on the inflammatory response, microglial activation in the mouse hippocampus was assessed by immunofluorescence labeling to determine the number, soma area, and process length of microglia. Iba1 immunofluorescence assessment demonstrated that LPS exposure significantly enhanced the number of microglia (**Figures 4A, B, D** **)** and activated microglia, as indicated by an increased soma area (**Figures 4A, B, E** **)** and shorter ramified processes (**Figures 4A, B, F** **)** compared with the control group. Interestingly, after pretreatment with rhFGF21 for 3 days, LPS-induced changes in microglial numbers and morphologies in the hippocampus were significantly reversed (**Figure 4**).

### Exogenous rhFGF21 Administration Reduced the Production of Proinflammatory Cytokines Induced by LPS Through Inhibiting NF-κB Signaling Pathway

**Figure 5:**
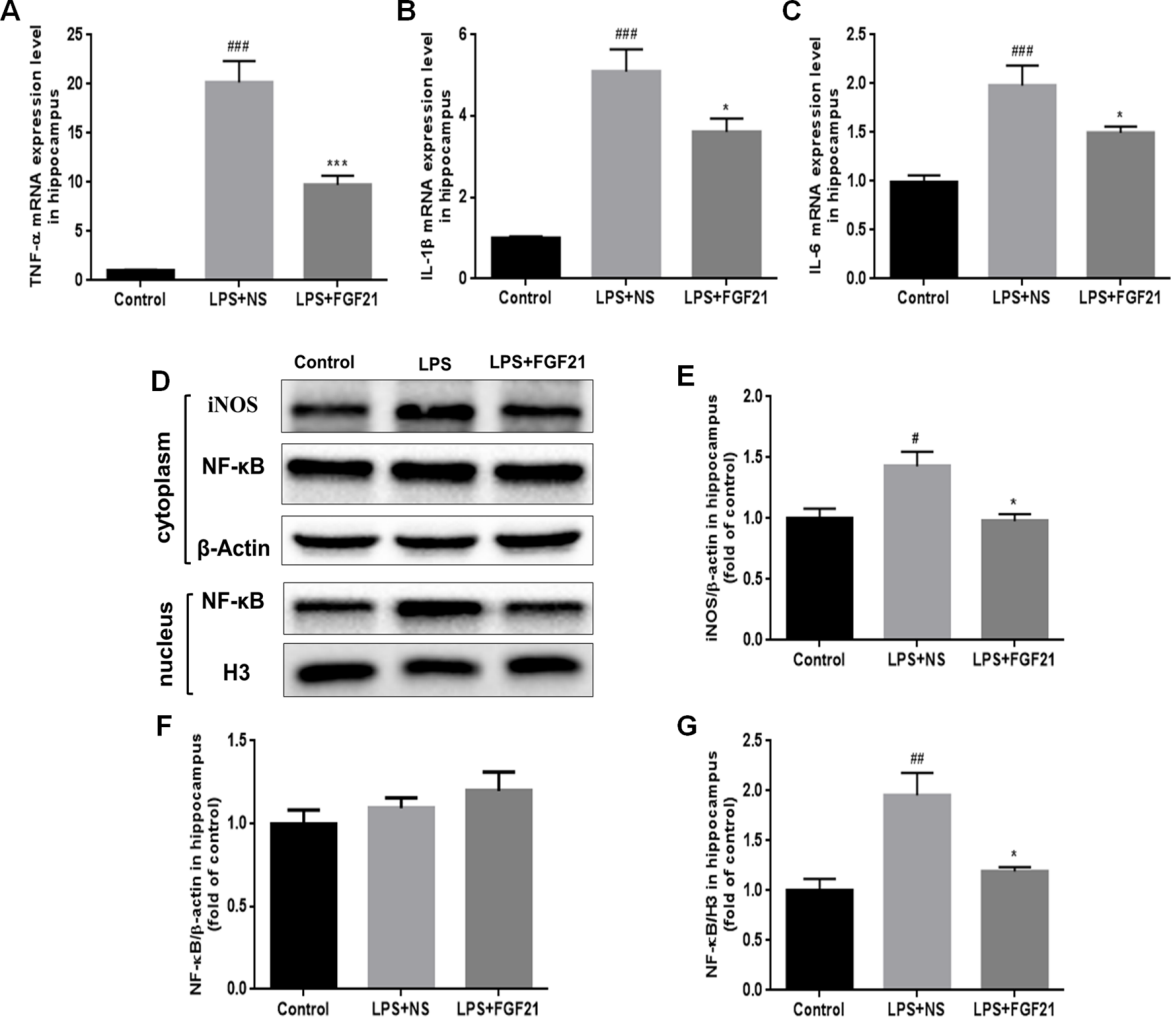
Exogenous rhFGF21 administration reduced levels of proinflammatory cytokines TNF-α, IL-1β and IL-6 induced by LPS through inhibiting NF-κB signaling pathway in the mouse hippocampus. **(A)** rhFGF21 decreased LPS-enhanced hippocampal pro-inflammatory cytokine TNF-α. **(B)** rhFGF21 decreased LPS-enhanced hippocampal pro-inflammatory cytokine IL-1β. **(C)** rhFGF21 decreased LPS-enhanced hippocampal pro-inflammatory cytokine IL-6. **(D)** Representative images of pro-inflammatory cytokine iNOS and NF-κB expression in cytoplasm and nucleus detected by western blot. **(E)** Densitometric analysis for the protein expression of iNOS in cytoplasm. **(F)** Densitometric analysis for the protein expression of NF-κB in cytoplasm. **(G)** Densitometric analysis for the protein expression of NF-κB in nucleus. Data are means ± SEM (n=5). ^#^P < 0.05, ^##^P < 0.01, ^###^P < 0.001 compared to control group; ^*^P < 0.05, ^***^P < 0.001 compared to LPS-induced group.

Microglial activation can induce the expression of inflammatory cytokines. Therefore, the expression levels of the critical proinflammatory cytokines IL-1β, TNF-α, and IL-6 were measured by RT-PCR, and iNOs expression was measured by western blotting. LPS exposure significantly triggered the mRNA expression of cytokines in the hippocampus, as indicated by the enhanced release of TNF-α, IL-1β, and IL-6. Conversely, rhFGF21 administration markedly reversed LPS-induced changes in the expression levels of these inflammatory cytokines (**Figures 5A–C**). As shown by western blot analysis, LPS administration significantly increased the production of iNOS, and pretreatment with rhFGF21 considerably reversed this increase (**Figures 5D, E** **)**.

The expression of inflammatory factors in LPS-triggered activated glial cells is regulated by NF-κB signaling pathways (Chen G. et al., 2018; Muhammad et al., 2019). LPS activated the NF-κB signaling pathway, as reflected by the increased nuclear level of NF-κB protein. To investigate whether rhFGF21 can decrease proinflammatory cytokine expression through inhibiting the NF-κB signaling pathway, cytoplasmic and nuclear proteins were extracted, and NF-κB was measured by western blotting with β-actin and H3 used as cytoplasmic and nuclear housekeeping proteins, respectively. There were no significant differences in NF-κB expression levels in the cytoplasm of cells in the LPS and rhFGF21 pretreatment groups (**Figures 5D, F** **)**. However, LPS treatment markedly increased nuclear NF-κB, and this increase was markedly reversed by pretreatment with rhFGF21 for 3 days (**Figures 5D, G** **)**.

### Exogenous rhFGF21 Could Function Through FGFR1 Activation

**Figure 6:**
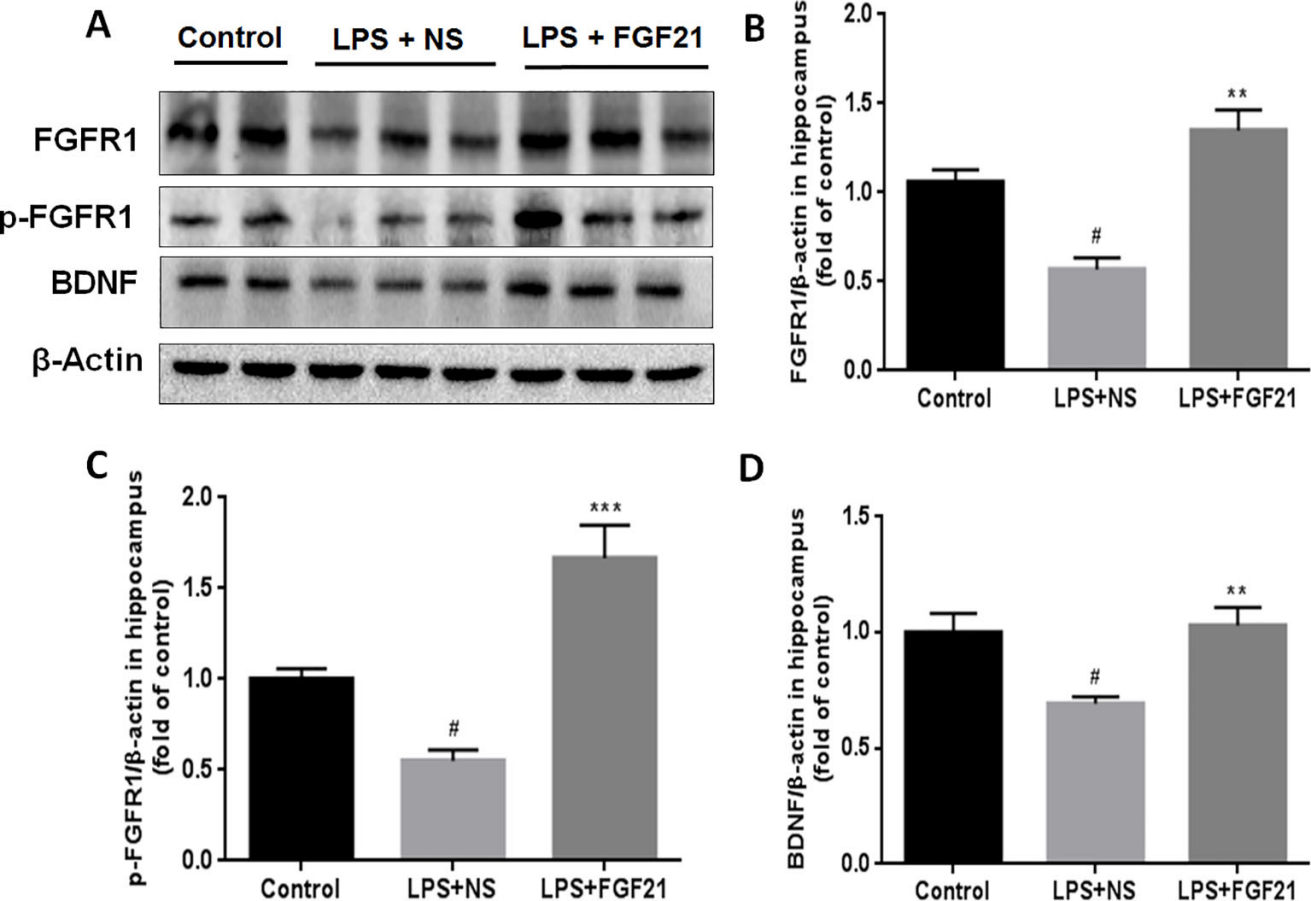
Exogenous rhFGF21 supplement functioned through FGFR1 activation and enhanced BDNF deficiency induced by LPS. **(A)** Representative images of FGFR1, p-FGFR1, and BDNF expression in the mouse hippocampus detected by western blot. **(B)** Densitometric analysis for the protein expression of FGFR1. **(C)** Densitometric analysis for the protein expression of p-FGFR1. **(D)** Densitometric analysis for the protein expression of BDNF. Data are means ± SEM (n=5). ^#^P < 0.05 compared to control group; ^**^P < 0.01, ^***^P < 0.001 compared to LPS-induced group.

FGFR1 is widely expressed throughout the nervous system and contributes to hippocampal nerve growth and long-term potentiation (Deng et al., 2019). To investigate whether the effect of rhFGF21 on depression is mediated through the FGFR1 signaling pathway, we detected the expression levels of FGFR1 and p-FGFR1 by western blotting. LPS administration markedly reduced FGFR1 and p-FGFR1 levels in the hippocampus compared with those in the control group, and rhFGF21 treatment significantly rescued this decrease in hippocampal FGFR1 and p-FGFR1 (**Figures 6A–C**).

BDNF is a major representative of the brain neurotrophic factors, which are involved in cognition control. Cognitive dysfunction in depressive patients is due to decreased levels of BDNF (Domingues et al., 2018), and BDNF has been suggested to influence the response to antidepressant treatment (Hennings et al., 2019). Therefore, the effect of rhFGF21 on BDNF expression was measured. LPS treatment significantly reduced BDNF expression compared with that in the control group. Interestingly, rhFGF21 prevented the decrease in BDNF levels in the mouse hippocampus induced by LPS administration (**Figures 6A, D** **)**.

### Exogenous rhFGF21 Administration Inhibited Inflammatory Cytokines Expression Through NF-κB Inhibition Mediated by FGFR1 Activation in Cultured Primary Microglia

**Figure 7:**
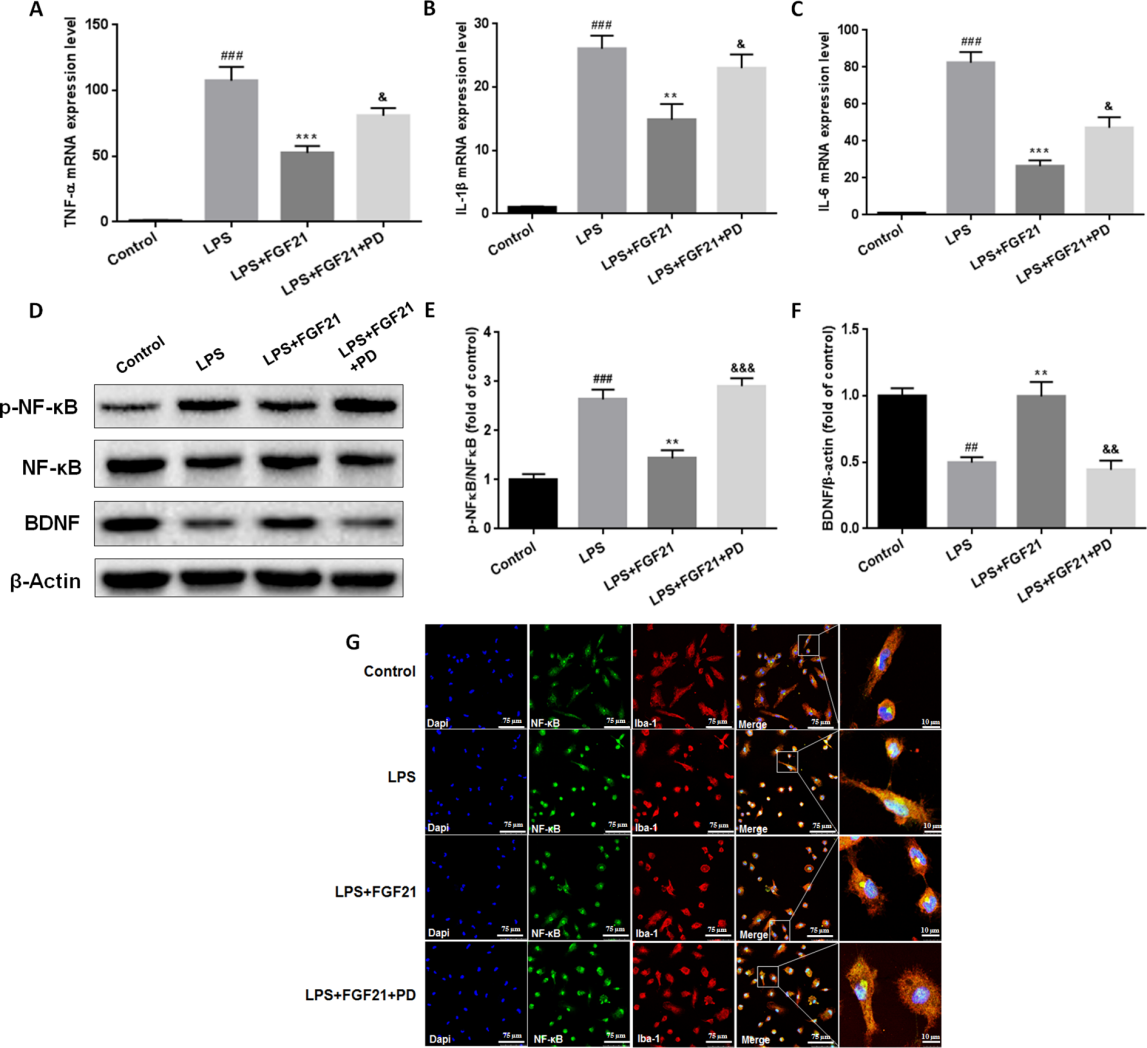
Exogenous rhFGF21 supplement suppressed pro-inflammation cytokines expression by activating FGFR1 through inhibiting phosphorylation of NF-κB, and ameliorated BDNF deficiency induced by LPS in primary microglia cells. **(A)** rhFGF21 administration suppressed pro-inflammation cytokines TNF-α mRNA level. **(B)** rhFGF21 administration suppressed pro-inflammation cytokines IL-1β mRNA level. **(C)** rhFGF21 administration suppressed IL-6 mRNA level. **(D)** Representative bands of NF-κB, p-NF-κB, and BDNF expression detected by western blot. **(E)** Densitometric analysis for the protein expression of p-NF-κB/NF-κB. **(F)** Densitometric analysis for the protein expression of BDNF. Data are means ± SEM (n = 5). ^##^P < 0.01, ^###^P < 0.001 compared to control group. ^**^P < 0.01, ^***^P < 0.001 compared to LPS treated group. ^&^P < 0.05, ^&&^P < 0.01, ^&&&^P < 0.001 compared to LPS and rhFGF21 co-treated group.

To further evaluate the effects of rhFGF21 on the inflammatory response of microglia and the underlying possible mechanism of action of these effects, primary microglia were extracted and isolated from the cerebral cortices of 1–2-day-old neonatal Sprague-Dawley rat pups. Meanwhile, since FGF21 usually functions by activating its receptor, to further investigate the function of FGFR1, its receptor, the FGFR1-specific inhibitor PD173074 (10 nM) was applied. Levels of the inflammatory cytokines IL-1β, TNF-α, and IL-6 were assessed by RT-PCR. The mRNA levels of IL-1β, TNF-α, and IL-6 were higher in the primary microglia of the LPS-treated group compared with the control group, while rhFGF21 treatment significantly reduced IL-1β, TNF-α, and IL-6 upregulation induced by LPS. Additionally, PD173074 significantly reversed the rhFGF21 treatment-induced downregulation of TNF-α, IL-1β, and IL-6 mRNA (**Figures 7A–C**), indicating that rhFGF21 suppresses inflammatory cytokine expression through activating FGFR1 in microglia.

**Figure 8:**
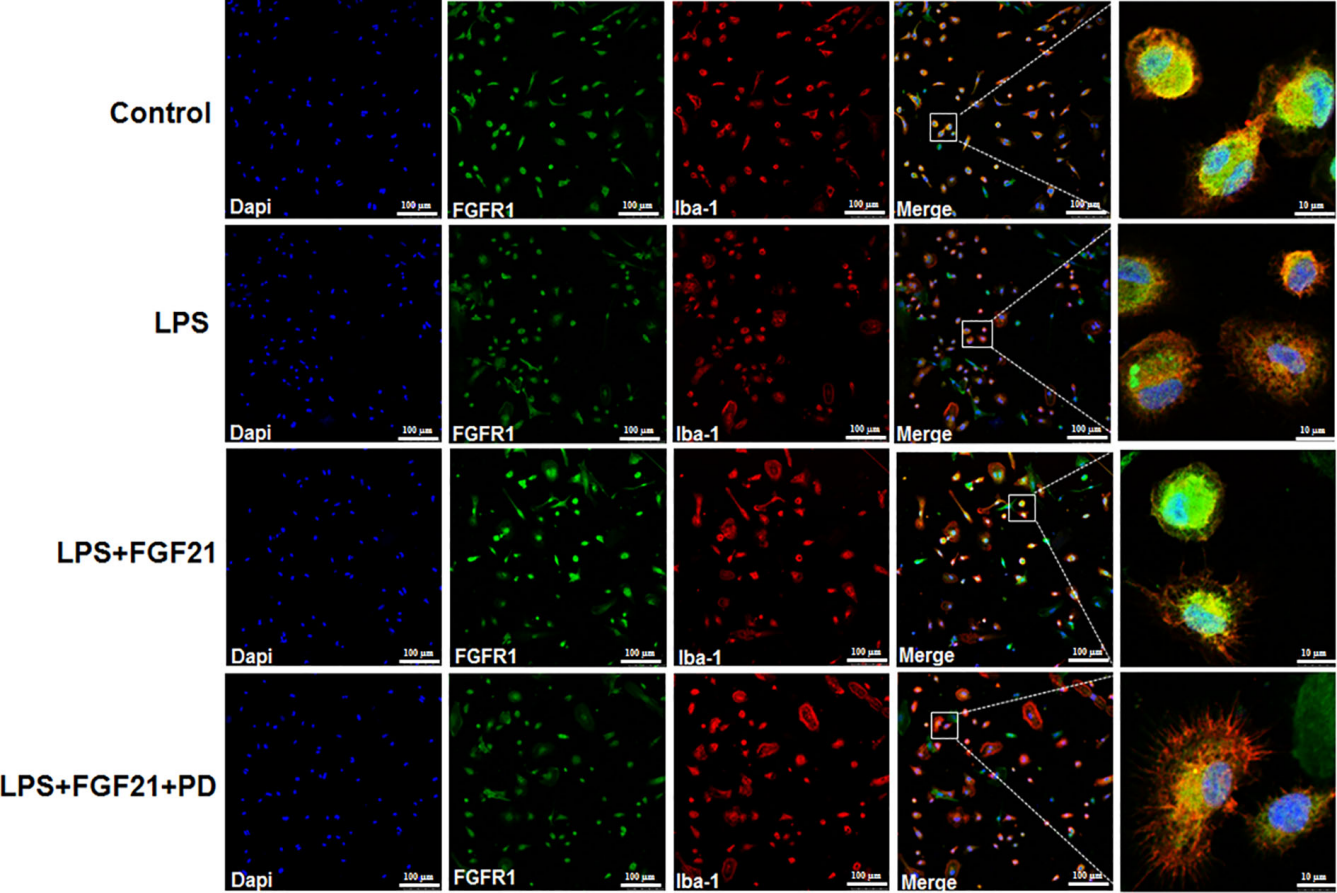
Exogenous rhFGF21 rescued LPS-induced downregulated FGFR1 activity detected by immunofluorescence staining.

NF-κB signaling pathway activation is involved and critical to the development of depression as it triggers the production of proinflammatory mediators (Su et al., 2017). To further explore the anti-depressive effects mediated by the anti-inflammatory mechanism of rhFGF21 in microglia, the protein levels of p-NF-κB and NF-κB in cultured primary microglia were analyzed by western blotting. The levels of p-NF-κB/NF-κB were significantly higher in the LPS-treated group than in the control group, and rhFGF21 could suppress NF-κB activation stimulated by LPS administration, while cotreatment with the FGFR1 inhibitor PD173074 and rhFGF21 significantly reversed the inhibitory effects of rhFGF21 on NF-κB (**Figures 7D, E** **)**. Consistent with the western blot data, LPS significantly stimulated NF-κB activation and translocated into nucleus. Exogenous rhFGF21 treatment obviously suppressed this translocation. Whereas PD173074 co-administration significantly reversed the effects of rhFGF21 on NF-κB (**Figure 7G**). Besides, the effect of rhFGF21 on BDNF expression was also analyzed. Consistent with the results in the animal study, LPS administration significantly reduced the level of BDNF compared with that in the control group; however, rhFGF21 markedly upregulated BDNF expression, and as expected, the FGFR1 inhibitor PD173074 significantly reversed this upregulation of BDNF (**Figures 7D, F**). To further confirm whether rhFGF21 functions through its receptor FGFR1, the immunofluorescence staining of FGFR1 was also performed. Consistent with the result in the animal model, LPS markedly suppressed FGFR1 activation, rhFGF21 rescued FGFR1 activation. Nevertheless, this was reversed by PD173074 coadministration (**Figure 8**). In summary, these results indicate that rhFGF21 inhibits proinflammatory cytokine expression through NF-κB inhibition mediated by FGFR1 activation.

## Discussion

Studies have demonstrated the association of four FGFs, FGF2, FGF9, FGF21, and FGF22, with depression, and the correlation between these FGFs and depression has been well reviewed by Deng et al. (2019). FGF2 is a neurotrophic factor widely expressed in the CNS. Administration of a combination of the antidepressants olanzapine and fluoxetine upregulated FGF2 levels (Maragnoli et al., 2004). Furthermore, FGF2 alleviated depression-like behaviors in rats (Turner et al., 2008). Chronic FGF-2 infusion blocked deficits in the sucrose preference test (SPT) and FST caused by chronic unpredictable stress (Elsayed et al., 2012). FGF9 is expressed mainly by neurons in the brain (Garcès et al., 2000). Exogenous FGF9 administration was found to increase depression-like behaviors, and exogenous FGF9 knockdown in the dentate gyrus improved anxiety-like behavior in rats (Aurbach et al., 2015). The effect of FGF9 was found to be opposite that of FGF2, which may be due to the inverse relationship between FGF9 expression and FGF2 expression, and FGF2 and FGF9 may act as physiological antagonists to mediate emotionality and vulnerability in mood disorders (Aurbach et al., 2015). FGF22 is expressed mainly in the skin and brain, and the deletion of endogenous FGF22 induced depression-like behavior, as shown by the FST, TST, and SPT (Williams et al., 2016). Recent studies have reported that FGF21 possesses various pharmacological activities including its antioxidant activity, suppression of apoptosis induced by endoplasmic reticulum stress (Liang et al., 2017), and anti-inflammatory activity (Wang Q. et al., 2018; Zhang et al., 2019). FGF21 weakly binds heparin and can pass through the blood-brain barrier by simple diffusion (Hsuchou et al., 2007). rhFGF21 has been demonstrated to have potent therapeutic activity in various neurological disorders, such as Parkinson's disease (Makela et al., 2014), Alzheimer's disease (Amiri et al., 2018; Chen et al., 2019), and ischemic stroke (Jiang et al., 2018; Yang X. et al., 2018). In the current study, pretreatment with rhFGF21 at doses of 1.5 and 3.0 mg/kg significantly prevented the increase in immobility time induced by LPS administration in the FST and TST, indicating the antidepressant-like effect of this large molecule. Notably, the administration of rhFGF21 alone did not significantly alter behavior compared with the control group, demonstrating that rhFGF21 is effective under only disease conditions.

The central or peripheral administration of LPS, an endotoxin, results in depression in animal models. Therefore, LPS treatment is a validated approach to establish a depression model, and LPS-induced depression-like animal models are commonly used to assess the anti-depressive effects of molecules. Various studies have demonstrated LPS-induced behavioral impairments including increased immobile time in the FST and TST (Brites and Fernandes, 2015), which is consistent with the results of our study. Pretreatment with 1.5 mg/kg rhFGF21 for three days markedly prevented and ameliorated LPS-induced depression-like behaviors (the increased total distance traveled and number of line crossings revealed by the OFT and decreased immobility time revealed by FST and TST), indicating the potential antidepressant effects of rhFGF21.

An increasing number of studies have suggested that inflammation plays a crucial role in the process of MDD (Rush et al., 2016; Yang M. et al., 2018). The activation of microglia, a kind of macrophage in the brain, can trigger the expression of proinflammatory cytokines involved in the pathological development of neuroinflammation- and degeneration-related diseases. The hippocampus is a brain region with a high density of microglia. Some studies have shown that microglia is activated in neuroinflammation, contributing to the development of depression (Wang Q. et al., 2018). LPS administration induced depression-like behaviors, triggered microglial activation in the hippocampus, and upregulated proinflammatory cytokine expression (Brites and Fernandes, 2015). Activated microglia are the major source of proinflammatory cytokines, such as TNF-α, IL-1β, and IL-6, in the CNS (Yang M. et al., 2018). Preclinical and clinical studies have proposed that proinflammatory cytokines are associated with the pathogenesis of depression (Karson et al., 2013) and that an increase in pro-inflammatory cytokines contributes to depressive-like behaviors (Raison et al., 2006; Guo et al., 2019). In addition, various studies demonstrated increased serum levels of TNF-α, IL-1β, and IL-6 in patients with depression (Song et al., 2009; Brites and Fernandes, 2015). In the current study, rhFGF21 could inhibit the activation of microglia and reduce the expression levels of the proinflammatory cytokines TNF-α, IL-1β, and IL-6 in the hippocampus of a depressive-like mouse model induced by LPS administration and in primary microglia stimulated by LPS.

The NF-κB signaling pathway plays a critical role in the inflammatory response. Under normal conditions, NF-κB is bound to the inhibitor IκB, and exists in an inactive state in the cellular cytoplasm. However, once activated by an activator, such as LPS, NF-κB is phosphorylated to p-NF-κB, which is then released and translocated into the nucleus, where it promotes the expression of inflammatory cytokines (Luo et al., 2014; Yang M. et al., 2018). In the current study, we examined nuclear and cytoplasmic levels of NF-κB in the hippocampus of an LPS-induced depression mouse model and determined the expression levels of NF-κB and p-NF-κB in LPS-induced primary microglia to assess the effects of rhFGF21 on the NF-κB signaling pathway. rhFGF21 could reduce the increase in the ratio of p-NF-κB/NF-κB induced by LPS and decrease the enhanced level of nuclear NF-κB induced by LPS, indicating that rhFGF21 could inhibit NF-κB activation and translocation into the nucleus. These results indicated that rhFGF1 may exert an anti-inflammatory effect by mediating the NF-κB signaling pathway and that rhFGF1 is a novel antidepressant candidate.

FGF21 activity requires its binding to a receptor complex that consists of FGFR1 and the coreceptor β-Klotho (Min et al., 2018). Previous studies have demonstrated that FGF21/FGFR1 signaling plays a major role in increasing basal glucose uptake (Hsu et al., 2019), inducing macrophages through the Ox-LDL-induced inflammatory response (Wang N. et al., 2018), attenuating neuronal apoptosis in the penumbra of rats after permanent middle cerebral artery occlusion (MCAO) (Zheng et al., 2019), and protecting the blood-brain barrier by increasing tight junction and adhesion junction proteins after traumatic brain injury (Chen J. et al., 2018). In the current study, to further investigate the functions of rhFGF21 in suppressing the NF-κB pathway to inhibit microglial activation and proinflammatory cytokine expression mediated through FGFR1 activation, the FGFR1 inhibitor PD173074 and rhFGF21 were used to treat primary microglia stimulated with LPS. rhFGF21 activated FGFR1, which suppressed proinflammatory cytokine expression and reduced the ratio of p-NF-κB/NF-κB expression stimulated by LPS in primary microglia. In contrast, PD173074 blocked these inhibitory effects of rhFGF21, indicating that rhFGF21 suppresses the NF-κB pathway to inhibit microglial activation and proinflammatory cytokine expression mediated through FGFR1 activation. Proinflammatory cytokines can reduce the expression of BDNF, and neurotrophic factors are critical in the pathogenesis of depression and brain neuroplasticity (Lee and Kim, 2010). Several reports demonstrated that LPS administration induced inflammatory cytokine expression and reduced BDNF and other neurotrophin expression, which could cause cognitive dysfunction. BDNF levels are much higher in the hippocampus than in other brain structures due to the great biological importance of the hippocampus in memory maintenance and its involvement with emotions. In addition, our study demonstrated that rhFGF21 could prevent LPS-induced BDNF downregulation by increasing BDNF expression in the mouse hippocampus and in primary microglia, which was reversed by its combination treatment with PD173074.

## Conclusion

In conclusion, the current study provides evidence that rhFGF21 treatment improves LPS-induced depression-like behaviors and decreases proinflammatory cytokine expression. In addition, the current study demonstrates that rhFGF21 can act as an antidepressant upon hippocampal microglial stimulation through inhibiting activation of the NF-κB signaling pathway by activating FGFR1. Accordingly, the present study indicates that rhFGF21 could be a potent candidate drug for antidepressant therapy. However, the underlying mechanism by which rhFGF21 affects activated microglia and inflammatory factors requires further study.

## Data Availability Statement

The raw data supporting the conclusions of this article will be made available by the authors, without undue reservation, to any qualified researcher.

## Ethics Statement

All animal use and care protocol conformed to the Guide for the Care and Use of Laboratory Animals from the National Institutes of Health and was approved by the Animal Care and Use Committee of Wenzhou Medical University.

## Author Contributions

XW and LL designed the experiment and wrote the manuscript. LZ, JH, RG, and SY performed the animal experiments and analyzed the data. FL, DW, and KG performed the cells experiments and analyzed the data. YZ, AH, and XW supervised the study and contributed to the design of the study. All the authors read and approved the final version of the manuscript.

## Funding

This work was supported by the National Natural Science Foundation of China (No. 81771284, 81971180), Natural Science Foundation of Zhejiang Province (LQ19H090012) and Wenzhou Municipal Science and Technology Bureau Project (Y20160078).

## Conflict of Interest

The authors declare that the research was conducted in the absence of any commercial or financial relationships that could be construed as a potential conflict of interest.

The handling editor is currently organizing a Research Topic with one of the authors LL, and confirms the absence of any other collaboration.
